# Soy isoflavone glycitein protects against beta amyloid-induced toxicity and oxidative stress in transgenic *Caenorhabditis elegans*

**DOI:** 10.1186/1471-2202-6-54

**Published:** 2005-08-25

**Authors:** Astrid Gutierrez-Zepeda, Ross Santell, Zhixin Wu, Marishka Brown, YanJue Wu, Ikhlas Khan, Christopher D Link, Baolu Zhao, Yuan Luo

**Affiliations:** 1 Department of Biological Sciences, University of Southern Mississippi, Hattiesburg, MS 39406, USA; 2 Department of Human Sciences, Alcorn State University, Alcorn, MS 39096, USA; 3 National Center for Natural Products Research, School of Pharmacy, Oxford, MS 38655, USA; 4 Institute for Behavioral Genetic, University of Colorado, Boulder CO 80309, USA; 5 Laboratory of Visual Information Processing, Center of Brain & Cognitive Science, Institute of Biophysics, Academia Sinica, Beijing 100101, P.R. China; 6 Department of Pharmaceutical Science, School of Pharmacy, University of Maryland, Baltimore, MD 21201-1180, USA

## Abstract

**Background:**

Epidemiological studies have associated estrogen replacement therapy with a lower risk of developing Alzheimer's disease, but a higher risk of developing breast cancer and certain cardiovascular disorders. The neuroprotective effect of estrogen prompted us to determine potential therapeutic impact of soy-derived estrogenic compounds. Transgenic *C. elegans*, that express human beta amyloid (Aβ), were fed with soy derived isoflavones genistein, daidzein and glycitein (100 μg/ml) and then examined for Aβ-induced paralysis and the levels of reactive oxygen species.

**Results:**

Among the three compounds tested, only glycitein alleviated Aβ expression-induced paralysis in the transgenic *C. elegans*. This activity of glycitein correlated with a reduced level of hydrogen peroxide in the transgenic *C. elegans*. *In vitro *scavenging effects of glycitein on three types of reactive oxygen species confirmed its antioxidant properties. Furthermore, the transgenic *C. elegans *fed with glycitein exhibited reduced formation of β amyloid.

**Conclusion:**

These findings suggest that a specific soy isoflavone glycitein may suppress Aβ toxicity through combined antioxidative activity and inhibition of Aβ deposition, thus may have therapeutic potential for prevention of Aβ associated neurodegenerative disorders.

## Background

Estrogen, a natural steroid long associated with effects on the female reproductive system, also plays a role in the central nervous system (CNS) through binding estrogen receptors located in the brain [1,2]. It has been demonstrated that estrogen has neuroprotective and neurotrophic properties [1-9]. Epidemiological studies suggest that post-menopausal women using Estrogen Replacement Therapy (ERT) have a decreased risk of developing dementia [10-12]. However, the beneficial effect of ERT on dementia associated with Alzheimer's disease (AD) is yet inconclusive [13-15]. Although ERT alleviates the symptoms associated with menopause and has positive effects on bones, ERT in post-menopausal women has been linked to a higher incidence of uterine and breast cancer. Consequently, the Selective Estrogen Receptor Modulators (SERMs) compounds that exert tissue specific estrogenic effects may provide the benefits of ERT without the risks. A group of natural SERMs are the soy-derived phytoestrogens, which are structurally similar to estrogen [16], and may serve as an alternative to ERT [17-19].

Soybeans contain a large amount of isoflavones, including genistein (4', 5'7-trihydroxyisoflavone), daidzein (4', 7-dihydroxyisoflavone), glycitein (6-methoxydaidzein) and their glycosides [20]. Experimental evidence suggests that soy isoflavones possess many properties including estrogenic [16], antioxidant [21] hypocholesterolemic [22], and inhibition of cell proliferation and DNA synthesis [23,24]. Phytoestrogens exert estrogen agonist and antagonist characteristics [17], in part because of differential binding affinities for the estrogen receptor (ER) isoforms; with higher affinity for ERβ than for ERα. Areas of the brain responsible for cognitive function and susceptible to AD (basal forebrain, hippocampus, cerebral cortex), express higher levels of ERβ compared to ERα [25]. Thus, interest in these compounds has grown because they could be used as SERMs, to delay or prevent the cognitive decline associated with AD [3,26] without increasing the risk of developing cancer [27].

AD is widely recognized as a serious public health problem [28]. The clinical symptoms of AD begin with memory impairment that eventually progresses to dementia, a process postulated to be the consequence of selective degeneration of nerve cells in those brain regions critical for memory, cognitive performance and personality [29]. AD is characterized by the presence of amyloid beta peptide (Aβ_1–42_) aggregation and increased oxidative stress, both causing neuronal injury and death [30]. An "amyloid cascade" hypothesis states that accumulation of Aβ deposition initiates a series of downstream neurotoxic events, which result in neuronal dysfunction and death [31]. The strongest evidence supporting this hypothesis comes from molecular genetic studies. Patients with Down's Syndrome, a disease related to an extra copy of chromosome 21 containing the APP gene, develop AD with the formation of Aβ deposits, an early sign of brain lesion [32]. All familiar forms of AD (FAD)-linked mutations, in the APP gene or two presenilin genes (PS1 and PS2), result in increased production of Aβ_42_, which is the more amyloidogenic form [33]. Transgenic mice overexpressing the mutant APP develop Aβ-containing amyloid plaques similar to those found in AD. Furthermore, inducing toxicity and cognitive dysfunction by introducing Aβ into organisms that do not have endogenous Aβ [34, 56] provided "gain of function" evidence for the "amyloid hypothesis". In addition, other structure lesions including neurofibrillary tangles and AproE might contribute to an imbalance between Aβ production and clearance [31]. Therefore, modulation of Aβ production and clearance in the brain is one approach for treatment of AD.

In order to understand the neuroprotective mechanism of phytoestrogens, we performed several experiments using a transgenic *Caenorhabditis elegans *model expressing the human amyloid-beta peptide (Aβ_1–42_). The transgenic *C. elegans *exhibits β amyloid fluorescence staining similar to those observed in the human brain [34], along with a concomitant progressive paralysis phenotype [35]. Results of these experiments suggest that the neuroprotective effect of phytoestrogens is, at least in part, due to its antioxidative activity.

## Results

### 1. Glycitein alleviates Aβ-induced paralysis in the transgenic C. elegans

**Figure 1:**
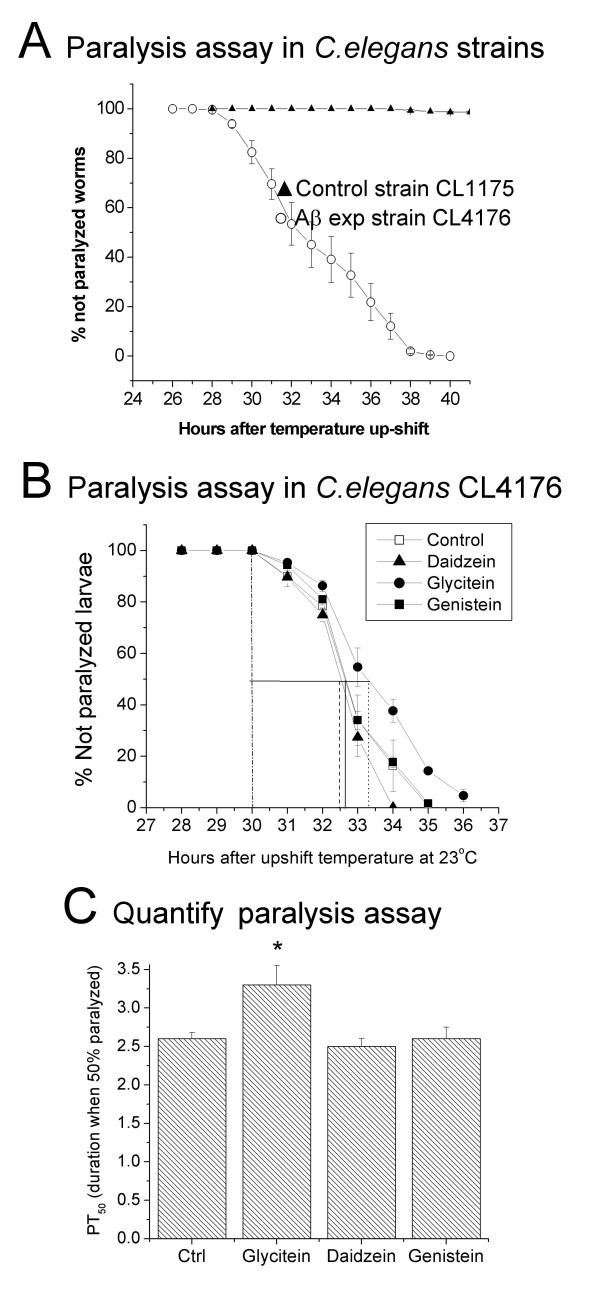
Paralysis assays in the transgnic *C. elegans*. **A**. Time course of paralysis in the transgenic strain CL4176 and the control strain CL1175. **B**. Paralysis assay in *C. elegans *CL4176 fed with different isoflavones. Synchronized eggs were maintained at 16°C for 38 h, on the 35 × 10 mm culture plates (~100 eggs/plate) containing vehicle (control), daidzein, glycitein, or genistein (100 μg/ml), followed by up-shifting the temperature to 23°C to induce the transgene expression. The paralysis was scored at 60 min intervals. Data are expressed as percentage of non-paralyzed worms from three independent assays of 100 worms in each experiment. **C**. The paralysis assays were quantitated for mean time duration at which 50% worms were paralyzed from 30 hrs after up shift temperature to 23°C (PT_50_). P values were obtained from 3 independent assays for the worms fed with different drugs each paired with untreated controls. Total 100 worms were used in each assay.

A relationship between the onset of Aβ expression and paralysis behavior has been established in the temperature-inducible transgenic *C. elegans *strain CL4176 [35]. We first conducted the paralysis assay using this strain to determine the effects of the isoflavones on Aβ-induced toxicity in the organism. We have observed in an independent study that the same transgenic *C. elegans *fed with *Ginkgo biloba *extract EGb 761, known for its antioxidant properties and beneficial effect for dementia, exhibited a delayed paralysis at the concentration ranging from 10 to 500 μg/ml, and this effect was not dose-dependent (data not shown). Age-synchronized *C. elegans *(CL4176, 100 worms/group) were fed with daidzein, glycitein, genistein or vehicle for 48 h prior to temperature up shift and then scored for paralysis. Figure 1A is a time course of a paralysis assay comparing a transgenic control strain CL1175, which does not express Aβ, with the Aβ-expressing strain CL4176 to demonstrate the specificity of Aβ-expression induced paralysis. Figure 1B and 1C represent paralysis in four groups of *C. elegans *CL4176 fed with one of the three different isoflavones (100 μg/ml) or vehicle. Apparently, Aβ-induced paralysis was delayed in worms fed with glycitein (Fig. 1B, filled circle compared with open squares, n = 3 assays, 100 worms/assay). Genistein, known to have more estrogenic activity than diadzein or glycitein [16], did not affect Aβ-induced paralysis in the nematode CL 4176 at the concentration applied (Fig. 1B filed squares, n = 3 assays, 100 worms/assay). The Aβ-induced paralysis was moderately accelerated at the end of the assay in the CL4176 worms fed with daidzein (Fig. 1B filled triangles, n = 3 assays, 100 worms/assay). Figure 1C shows a statistical analysis of the paralysis assays displayed in Fig. 1B. We define PT_50 _as time duration at which 50% worms were paralyzed from 30 hrs after up shift of temperature to 23°C. Statistically, a significant delay of Aβ-induced paralysis was only observed in the worms fed with glycitein (Fig. 1C, Control, PT_50 _= 2.6 ± 0.08 h vs. Glycitein, PT_50 _= 3.3 ± 0.25 h. p = 0.036; Daidzein, PT_50 _= 2.5 ± 0.10 h, p = 0.46; Genistein, PT_50 _= 2.6 ± 0.15 h. p = 0.76; n = 3 assays each drug, 40 worms in each assay group). Although Daidzein accelerated paralysis at the end point, PT_50 _did not indicate significant difference (Fig 1C) compared with that of the controls. It is known that the effective concentration for genistein to activate the estrogen receptor and tyrosine kinases is much lower (nM). Differential concentration effects of genistein might contribute to protection against Aβ toxicity/paralysis. Thus, we conducted experiments using genistein at two lower doses (10 μg/ml and 0.1 μg/ml). Aβ-induced paralysis was not affected in the worms fed with either of the two concentrations (data not shown), supporting the view that the effect of glycitein is specific.

To determine the overall effect of the isoflavones on the behavioral of the *C. elegans*, we conducted oxidative stress sensitivity assay and life span assay. We found that the *C. elegans *fed with glycitein were more resistant toward an oxidative stressor Juglone than the worms fed with daidzein and genistein (data not shown). However, the maximum life span was not affected in the *C. elegans *CL2006 fed with glycitein compared with untreated control worms (data not shown).

### 2. Glycitein attenuates levels of H_2_O_2 _in the Aβ-expressing C. elegans & in vitro

**Figure 2:**
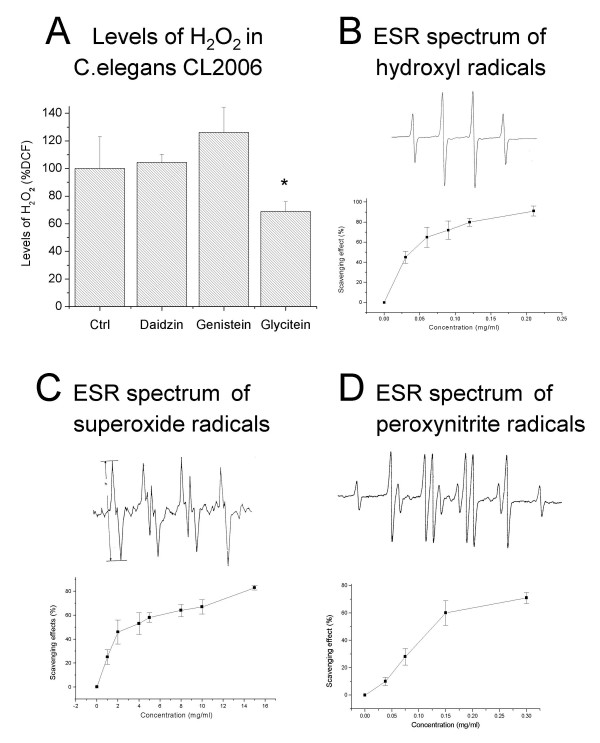
Scavenging effect of glycitein in the transgenic *C. elegans *and *in vitro*. **A**. H_2_O_2 _level in transgenic *C. elegans *CL4176 fed with different isoflavones. *C. elegans *strain CL4176 was maintained at 16°C for 38 h and then temperature up-shifted to 23°C for 48 h, followed by measurement of H_2_O_2 _(DCF assay described in methods). CL4176 worms were fed vehicle (Ctrl), 100 μg/ml daidzein, genistein or glycitein from 1 day of age until 3 days of age. At least 60 worms from each group were analyzed for levels of H_2_O_2_. Results are expressed as a percentage of fluorescence (%DCF) relative to control. **B**. Scavenging effect of glycitein on hydroxyl radicals *in vitro*. The ESR conditions: X-band, 100 kHz modulation with amplitude 1 G, microwave power 10 mW, central magnetic field 3,250 G, sweep width 200 G, temperature 20°C. Inset: ESR spectrum of DMPO-OH generated from Fenton reaction and trapped by DMPO. **C**. Scavenging effect of glycitein on superoxide radicals in the system. The ESR conditions are the same as in Fig. 3B. Inset: ESR spectrum of DMPO-OOH generated from Xanthine/xanthine oxidase and trapped by DMPO; **D**. Scavenging effect of Soy isoflavone glycitein on .CH_3 _free radicals in the in vitro system. Inset: ESR spectrum of CH_3_-tNB generated from the oxidation of DMSO by ONOO^-^and trapped by tNB.

Given that soy isoflavones are potent antioxidants, we determined whether the antioxidative properties of the isoflovones might contribute to protection against Aβ-toxicity. Previously, we established an *in vivo *assay for the measurement of intracellular H_2_O_2_-associated ROS in *C. elegans *[36]. The transgenic *C. elegans *were fed with or without the isoflavones, prior to induction of Aβ-expression, followed by measurement of the levels of H_2_O_2 _in the organism. Figure 2A demonstrates that the levels of ROS in the *C. elegans *CL2006 fed with glycitein for 36 h were reduced (control 100 ± 23%, glycitein 68.9 ± 7 %, n = 3, p = 0.05). Although genistein increased the levels of ROS compared with the untreated controls (Ctrl 100 ± 23 %, genistein 126.1 ± 18 %, n = 3, p = 0.28 total 300 worms in each group), it is not statistically significant. Daidzein did not affect Aβ-induced elevation of ROS (Ctrl 100 ± 23%, daidzein 104.4 ± 6%, n = 6, p = 0.74). These results suggest the decreased Aβ toxicity by glycitein might be, in part, a consequence of its antioxidative action.

To confirm the scavenging effect of glycitein on different species of oxidative free radicals *in vitro*, we first measured its effect on hydroxyl radicals. The hydroxyl free radicals were generated from Fenton reaction (H_2_O_2 _3%, FeSO_4 _0.1 mM and tapped by DMPO (0.1 mol/l). A spectrum with 4 lines and 1:2:2:1 intensity (g = 2.0045, a_N _= a_H _= 14.9 G) were obtained (Fig. 2B). Figure 2B demonstrates the signal intensity decrease with different concentrations of the soy isoflavone glycitein added into this system. The soy isoflavone glycitein appears to have very strong scavenging effects on hydroxyl radical generated from Fenton reaction (IC_50 _= 0.035 mg/ml).

We then determined the scavenging effect of glycitein on superoxide free radicals. The superoxide free radicals were generated from xanthine/xanthine oxidase and trapped by DMPO. A signal with 12 lines (a_N _= 14.2 G, a_H_^β ^= 11.2 G, a_H_^γ ^= 1.3 G) was obtained (Fig. 2C), and it was decreased with addition of glycitein as shown in Fig. 2C. Apparently, soy isoflavone glycitein has moderate scavenging effect on superoxide free radicals generated from the reaction of xanthine/xanthine oxidase (IC_50 _= 2 mg/ml).

The reaction of NO with superoxide free radicals is very fast (6.4 × 10^9^mol/L^-1^s^-1^) and forms peroxynitrite (ONOO^-^). In alkaline solution, it is stable but has a pKa of 6.6 at 0°C and decays rapidly once protonated, to hydroxyl radical-like species and NO_2_, which can oxidize sulfhydryls and membrane lipid causing cell toxicity and some diseases. To determine the scavenging effects of the soy isoflavone glycitein on ONOO^-^, the methyl free radical was generated from the oxidation of DMSO by ONOO^- ^and trapped by tNB and a spectrum with 12 lines (a_N _= 17.2 G, a_H _= 14.2 G) (Zhao et al. 1996) was obtained (Fig. 2D). A strong scavenging effect of glycitein on ONOO^- ^(IC_50 _= 0.13 mg/ml) was found as shown in Fig. 2D.

### 3. β amyloid were significantly reduced in transgenic C. elegans fed with glycitein

**Figure 3:**
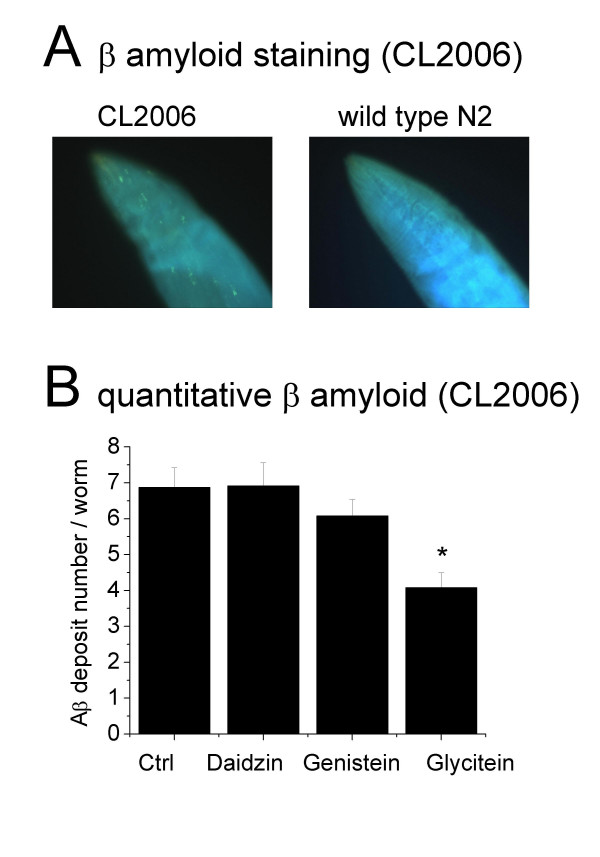
Effect of soy isoflavones on Aβ deposits in transgenic *C. elegans*. **A**. Representative images of thioflavin S staining in the transgenic (left) or wild type (right) worms. **B**. Quantitative Aβ formation. β amyloid were stained with thioflavin S in *C. elegans *CL2006 fed with or without isoflavones (100 μg/ml) for 4 days starting at the second day of age. β amyloid were examined using a fluorescence microscope. The quantity of β amyloid is expressed as mean number of Aβ deposits/worm head area (n = 24).

The modified "amyloid hypothesis" states that Aβ-induced oxidative stress may speed up β amyloid formation and lead to neuronal cell death in AD [37]. To determine whether soy isoflavones affect β amyloid formation *in vivo*, we measured β amyloid in the transgenic *C. elegans *CL2006 by thioflavin S staining. β amyloid was stained and the fluorescent images were quantified. Quantitatively (Fig. 3), the mean numbers of β amyloid staining per head area of the nematode are significantly reduced only in the transgenic *C. elegans *(CL2006) fed with glycitein (4.1 ± 0.4) compared with unfed controls (6.9 ± 0.5). A moderate reduction, although not significant, was observed in the *C. elegans *fed with genistein (6.1 ± 0.5). No change of Aβ deposits was observed in the worms fed with daidzein (6.9 ± 0.6). None of the three soy isoflavones inhibited Aβ aggregation in vitro (data not shown), suggesting that the decreased β amyloid by glycitein in the transgenic *C. elegans *(Fig. 1) is not due to its direct binding to Aβ, but might be a consequence of its antioxidative action (Fig. 2).

## Discussion

In this study, we employed a transgenic *C. elegans *model to evaluate the pharmacological effect of the soy-derived isoflavones genistein, glycitein and daidzein, on Aβ-initiated toxicity and oxidative stress. Results of these assays indicate that among the three isoflavones tested, glycitein delayed Aβ induced paralysis and attenuated the levels of amyloid formation in the transgenic *C. elegans*. In addition, glycitein significantly scavenged hydroxyl free radicals and inhibited the oxidation of peroxynitrite *in vitro*.

There has been strong evidence for the neuroprotective role of estrogen in aging animal studies and human studies [8,11,26,38-42]. Evidence for estrogens effect on cognition in women with AD is controversial [10,14]. However, it was reported that ovariectomized guinea pigs had a pronounced accumulation of β-amyloid plaques compared to intact controls and that estrogen replacement reversed the accumulation [3]. A proposed mechanism for estrogen inhibition of plaque formation is that estrogen induces the cleavage of membranous amyloid precursor protein (APP) generating a soluble proteolytic fragment that precludes the development of β-amyloid plaque formation [5,6]. The possible link between estrogen and Aβ prompted us to determine the effect of phytoestrogens on Aβ-induced toxicity in a model organism. Knowing that *apl-1*, the member of APP family in *C. elegans*, lacks a recognizable Aβ sequence [57, 58], the effect of phytoestrogens may have different mechanisms of action. Phytoestrogens have received increasing attention due to their potential protective effects against age-related diseases and hormone-dependent cancers. Phytoestrogens have the ability to selectively activate estrogen receptors, thus affecting many of the biological responses that are caused by endogenous levels of estrogen without concurrent and undesired side effects. Phytoestrogens may act both as an agonist and antagonist in a tissue specific manner [4]. It was suggested that phytoestrogenes can significantly influence sexually dimorphic cognitive behavior by enhancing spatial memory in young adult female animals but inhibit this ability in male [4].

Our observation that glycitein, with weaker estrogenic activities than genistein and daidzein, inhibited Aβ-induced paralysis and deposition, suggests that neuroprotection by phytoestrogens may not be mediated through the estrogenic activity of the compounds. Compared to other soy isoflavones, the estrogenic activity of glycitein is 20 times lower than genistein and daidzein and 200 times lower than 17β estradiol [16]. Soybeans contain large amounts of glycitein and its glycosides, which have been reported to inhibit growth and DNA synthesis of smooth muscle cells [23].

Apparently, it is the antioxidant activity that contributed to the protective effect by glycitein against Aβ-toxicity (Fig. 1) since glycitein is the only soy isoflavone which significantly attenuated the levels of ROS in the *C. elegans *(Fig. 2). Oxidative free radicals have been postulated as a cause of aging and of some degenerative diseases [45,46]. The formation of free radicals by Aβ *in vitro *[46] and profound induction of protein carbonyl in the transgenic *C. elegans *suggests that Aβ-induced oxidative stress triggers Aβ-induced paralysis in the *C. elegans *[47]. Although Aβ aggregations have been identified as neurotoxic to the brain, oxidative stress is predicted to occur before these aggregations [47] leading to cell apoptosis. Thus the observed reduction in amyloid formation might also be due to the anti-oxidant activities of glycitein. These observations go along with the free radical hypothesis of aging, which states that there is an imbalance of free radicals and reactive oxygen species (ROS) in the brain causing significant damage to key cellular components [45]. This imbalance may be the causative agent for the pathology of neurodegenerative disorders (such as AD) since most of these disorders are associated with age [48]. The toxicity of free radicals depends on the kinetics of their production, as well as on their stability and transfer efficiency to lipids and proteins. These radicals may interact with other radicals to produce Aβ aggregates [49], and promote the cleavage of the Aβ precursor (APP) supporting the idea that AD can be attributed to continuous oxidative stress, along with a weakened antioxidant status [49].

The causal relationship between ROS and Aβ has been long debated in the field. The transgenic *C. elegans *would allow us to address the issue. We have conducted a paralysis assay in the *C. elegans *fed with vitamin C and EGb 761, a *Ginkgo biloba *leaf extract. Surprisingly, vitamin C alone did not delay Aβ-induced paralysis, but it did when combined with EGb 761, which also inhibits Aβ oligomerization (data not shown), suggesting that it is the combined actions of antioxidants and other protection against Aβ toxicity that is necessary for alleviating Aβ-induced paralysis. Thus, we consider that the antioxidant action is only partially contributing to the protection against Aβ toxicity. Same argument may apply to the discrepancy of the genistein's effect between Fig 2A and Fig 3B; the increased levels of ROS by genistein did not correlate with a decreased Aβ deposition. Defining a functional relationship between Aβ deposition and toxicity, and ROS level is certainly one of our future directions.

The assumption that the protective effect by glycitein against Aβ toxicity might not be mediated by its action on the estrogen receptor is supported by our observation that genistein, with strongest estrogenic activity among soy isoflavones, did not offer protection again Aβ-toxicity. Genistein is a known tyrosine kinase inhibitor. The effective concentration for genistein to activate the estrogen receptor and inhibit tyrosine kinases is much lower (nM-μM) than the concentration we applied to the worms [4] and [5]. These differential concentration effects of genitein might offer protection against Aβ toxicity/paralysis. However, our additional experiments using much lower dosage of genistein did not provide evidence to support this notion. Aβ-induced paralysis was not affected in the worms fed with either of the two lower concentrations. Since at the given concentration (10 μg/ml, i.e. 37 μM), we observed effects with glycitein but not genistein, we assume that they have differential effects on Aβ-induced paralysis. It has been shown that high dose of genistein (μM) could cause apoptosis in rat primary cortical neurons *in vitro *via a calcium dependent pathway [43].

We demonstrated a consistent, correlative effect by glycitein against Aβ-induced toxicities using different assays, which suggests that *C. elegans *is a valid model for mechanistic examination of the transgene products as well as for pharmacological analysis of time course and kinetics of drug effect [50,51]. A relationship between Aβ amino-acid sequence, amyloid formation and oxidative damage was established using this model. Yatin et al. [46] showed both *in vitro *and in the *C. elegans *model that methionine (Met^35^) is critical for free radical production by Aβ_1–42_, and it is also critical for β-sheet formation in the transgenic *C. elegans *lines [52]. A correlation between a progressed paralysis phenotype with increased levels of protein carbonyls in CL4176 [47] supports the advanced "amyloid hypothesis" [37]. Mammalian αB-crystallin (CRYAB) a stress-inducible chaperone protein, which inhibits fibril formation of Aβ-(1–42) [53], has a protein homologue HSP-16 in the C. elegans. This protein has been reported to be colocalized with intracellular Aβ and up regulated in the transgenic Aβ-expression strain of C. elegans [35]. We previously demonstrated that a neuroprotectant, EGb 761, an extract from the *ginkgo biloba *tree leave, suppressed HSP-16 expression [54]. Although many protein molecules including estrogenic receptors are conserved in the nematode [55], the lack of correlation between isoflavone estrogenic activity and suppression of Aβ toxicity in this model system may not exclude the neuroprotection estrogen in AD patients. Nevertheless, it is likely that the temporal sequence of events manifested in the transgenic worms is the same as the one demonstrated in a *Drosophila *model of AD [56] in that accumulation of Aβ_42 _in the brain is sufficient to cause cognitive impairment and neurodegeneration.

## Conclusion

We used a transgenic *C. elegans *model to evaluate the pharmacological effect of the soy-derived isoflavones genistein, glycitein and daidzein, on Aβ-initiated toxicity and oxidative stress. Among the three compounds tested, only glycitein alleviated Aβ expression-induced paralysis in the transgenic *C. elegans*, which correlated with a reduced level of hydrogen peroxide and β amyloid. These findings suggest that the neuroprotective effect of phytoestrogens is probably due, at least in part, to its antioxidative activities.

## Methods

Soy isoflavones were obtained from the National Natural Products Research Center (Oxford, MS). Stock solutions of the soy isoflavones (1 mg/ml, 1000× stock solution) were made in 100% ethanol. The final concentration of ethanol in the food did not exceed 0.01%. DMPO (5,5-dimethyl-1-pyroline-1-oxide, tNB(3,3,5,5-tetramethyl-pyrroline N-oxide) were purchased from Sigma Chem Co. DMPO was purified by active charcoal.

### C. elegans strains

The construction and characterization of the transgenic nematode strains CL2006 and CL4176 have been described previously [34,35]. The CL2006 strain constitutively produces a muscle-specific Aβ_1–42_, whereas the expression of Aβ_1–42 _in CL4176 depends on a temperature up-shift from 16 to 23°C. Age-synchronized wild type (N2) and the transgenic CL2006 were propagated at 20°C in a temperature-controlled incubator (Sheldon Manufacturing, Model 2005, Cornelius, OR), CL4176 at 16°C, on solid nematode growth medium (NGM) seeded with *E. coli *(OP50) for food. All chemicals for treatment of experimental animals were added directly to the OP50 food source and began when larvae were 2 days old (for CL2006). In most cases, the nematodes were treated for 4 days (after hatching) with their respective drug. In the life span assay, the *C. elegans *were treated with the drug for the duration of their lifetime.

### Paralysis assays

C. elegans strain CL4176 [35,47] was maintained at 16°C and egg-synchronized onto 35 × 10 mm culture plates containing vehicle or drug. The worms (100 worms on each plate) were allowed to grow for 38 h at 16°C. After 38 hours the temperature was up shifted to 23°C to induce Aβ expression. Paralysis was scored at 1 h intervals until all worms were paralyzed

### H_2_O_2 _assay in C. elegans

Intracellular levels of H_2_O_2_-related reactive oxidative species (ROS) were measured in *C. elegans *using 2,7-dichlorofluorescein diacetate (DCF-DA; Molecular Probes). At the end of the specified treatment times, the *C. elegans *were collected into 100 μl phosphate buffered saline (PBS) (molarity) with 1% Tween-20 (PBST) in eppendorf tubes. The worms were then sonicated (Branson Sonifier 250, VWR Scientific, Suwanee, GA) and pipetted into wells of 96-well plates containing DCF-DA (final concentration 50 μM in PBS). Samples were read every 10 min for 2.5 h. in an FLx800 Microplate Fluorescent Reader (Bio-Tek Instruments, Winooski, VT) at 37°C at excitation 485 nm and emission 530 nm.

### ESR assay of free radicals

In order to measure the effect of glycitein on free radicals, the spin trap and the system-generated free radicals were mixed and measured with ESR spectrometer and the signal intensity was taken as Ho. Then the system with addition of glycitein was measured again. Hydroxyl radicals (H_2_O_2 _3%), Fe_2_SO_4 _(0.1 mM) and DMPO (0.1 mol/l) were mixed and sucked into a quartz capillary for ESR measurement, and the signal intensity was taken as Hx. The scavenging effect was calculated by [(Ho-Hx)/Ho] × 100%. The ESR spectra were recorded with Brucker ER200 D-SRC ESR spectrometer. Parameters were employed as follows: X-band, 100 kHz modulation with amplitude 1 G, microwave power 10 mW, central magnetic field 3,250 G, sweep width 200 G, temperature 20°C.

### Fluorescent staining and quantitation of β amyloid

Individual CL2006 transgenic nematodes were fixed in 4% paraformaldehyde/PBS, pH 7.4, for 24 h at 4°C, and then permeabilized in 5% fresh β-mercaptoethanol, 1% Triton X-100, 125 mM Tris pH 7.4, in a 37°C incubator for 24 h. The nematodes were transferred, stained with 0.125% thioflavin S (Sigma) in 50% ethanol for 2 min, destained for 2 min in 50% ethanol, washed with PBS and mounted on slides for microscopy. Fluorescence images were acquired using a 40× objective of a fluorescence microscope (BX 60, Olympus, Tokyo, Japan) equipped with a digital camera (Micropublisher 5.0, QIMAGING, Burnaby BC, Canada). The Thioflavin S-reactive deposits anterior of the pharyngeal bulb in individual animals were scored.

### Statistical analyses

All statistical tests were performed using a PC-based version of the statistical program Origin 6.0 software (Microcal Software, Inc., Northampton, MA). Statistical comparisons between treatments were done with unpaired student t-test. All figures indicate means and standard error of the mean. Differences with a *p *value less than 0.05 were defined as statistically significant.

## List of abbreviations used

AD, Alzheimer's disease

ROS, reactive oxygen species

H_2_O_2_, hydrogen peroxide

Aβ, beta amyloid peptide

ERT, Estrogen Replacement Therapy

SERMs, Selective Estrogen Receptor Modulators

## Authors' contributions

AGZ carried out the paralysis assay, the oxidative stress assay and measurement of levels of ROS. MB conducted some of the oxidative stress assays. ZW performed the fluorescence staining for Aβ deposits and the qantitation; JW performed additional experiments for the revision; IK provided the soy isoflavones; CL generated the transgenic C. elegans; BZ participated in the design and analysis of the experiments regarding *in vitro *assay of ROS; YL participated in the general design of the study, organized collaboration as well as finalizing the manuscript. All authors have read and approved the final manuscript.
